# CRISPR/Cas9-Mediated Disruption of the *pcz1* Gene and Its Impact on Growth, Development, and Penicillin Production in *Penicillium rubens*

**DOI:** 10.3390/jof9101010

**Published:** 2023-10-13

**Authors:** Carlos Gil-Durán, Diego Palma, Yudethzi Marcano, José-Luis Palacios, Claudio Martínez, Juan F. Rojas-Aedo, Gloria Levicán, Inmaculada Vaca, Renato Chávez

**Affiliations:** 1 Departamento de Biología, Facultad de Química y Biología, Universidad de Santiago de Chile (USACH), Santiago 9170022, Chile; cagild@gmail.com (C.G.-D.); yudethzi.marcano@usach.cl (Y.M.); juan.rojasaed@usach.cl (J.F.R.-A.); gloria.levican@usach.cl (G.L.); 2 Departamento de Química, Facultad de Ciencias, Universidad de Chile, Santiago 7800003, Chile; diegopalma.98@gmail.com; 3 Centro de Estudios en Ciencia y Tecnología de los Alimentos (CECTA), Universidad de Santiago de Chile (USACH), Santiago 9170022, Chile; jose.palacios@usach.cl (J.-L.P.); claudio.martinez@usach.cl (C.M.); 4 Departamento de Ciencia y Tecnología de los Alimentos, Facultad Tecnológica, Universidad de Santiago de Chile (USACH), Santiago 9170022, Chile

**Keywords:** Zn(II)_2_Cys_6_ protein, *pcz1*, CRISPR-Cas9, penicillin production, asexual development, *Penicillium rubens*

## Abstract

*Penicillium rubens* is a filamentous fungus of great biotechnological importance due to its role as an industrial producer of the antibiotic penicillin. However, despite its significance, our understanding of the regulatory mechanisms governing biological processes in this fungus is still limited. In fungi, zinc finger proteins containing a Zn(II)_2_Cys_6_ domain are particularly interesting regulators. Although the *P. rubens* genome harbors many genes encoding proteins with this domain, only two of them have been investigated thus far. In this study, we employed CRISPR-Cas9 technology to disrupt the *pcz1* gene, which encodes a Zn(II)_2_Cys_6_ protein in *P. rubens*. The disruption of *pcz1* resulted in a decrease in the production of penicillin in *P. rubens*. This decrease in penicillin production was accompanied by the downregulation of the expression of *pcbAB*, *pcbC* and *penDE* genes, which form the biosynthetic gene cluster responsible for penicillin production. Moreover, the disruption of *pcz1* also impacts on asexual development, leading to decreased growth and conidiation, as well as enhanced conidial germination. Collectively, our results indicate that *pcz1* acts as a positive regulator of penicillin production, growth, and conidiation, while functioning as a negative regulator of conidial germination in *P. rubens*. To the best of our knowledge, this is the first report involving a gene encoding a Zn(II)_2_Cys_6_ protein in the regulation of penicillin biosynthesis in *P. rubens*.

## 1. Introduction

The genus *Penicillium* is one of the largest and most diverse in the fungal kingdom. Most species in this genus are saprophytic, displaying high catabolic activity and the ability to produce a wide range of specialized metabolites [1]. Moreover, several species within the *Penicillium* genus are economically important. One of these species is *Penicillium rubens*. This filamentous fungal species is an important industrial producer of β-lactam antibiotics, specifically penicillins, and it comprises historically important penicillin-producing strains formerly classified as *Penicillium chrysogenum* [2]. Penicillins are of great economic importance, serving as both direct therapeutic agents against certain diseases and as a scaffold for the development of semi-synthetic antibiotics [3].

At the molecular level, the biosynthesis pathway of penicillin in *P. rubens* was elucidated many years ago and has been extensively documented in recent reviews [3,4,5]. The production of this specialized metabolite involves the sequential action of three enzymes encoded by the genes *pcbAB*, *pcbC*, and *penDE*, which form a biosynthetic gene cluster (BGC) [3,4]. These enzymes, namely δ(α-aminoadipyl)-cysteinyl-valine (ACV) synthetase, isopenicillin N synthase, and isopenicillin N acyltransferase, synthesize penicillin from the precursor amino acids α-aminoadipate, cysteine, and valine [3,4].

The regulation of penicillin biosynthesis in *P. rubens* involves a complex network of regulatory signals, transduction pathways, and effector proteins [3,4,6,7]. Interestingly, the penicillin BGC lacks a gene encoding for a specific transcription factor [8,9]. Thereby, the production of this metabolite in *P. rubens* is governed by well-known global regulators in fungi, including CreA, AreA, PacC, LaeA, Velvet proteins, PcRFX1, and PcFKH1 [3,4,6].

Zinc finger proteins constitute one of the largest families of regulators in eukaryotes. These proteins are classified into different groups based on the type of “zinc fingers” they possess [10,11]. Among these proteins, those containing a Zn(II)_2_Cys_6_ domain, in which six cysteines coordinate two zinc atoms, are particularly interesting in fungi, where they are exclusively found [10,11]. Fungal genomes can contain hundreds of genes encoding Zn(II)_2_Cys_6_ proteins, and several of them have been functionally characterized in diverse genera such as *Aspergillus*, *Bipolaris*, *Fusarium*, *Trichoderma*, and *Penicillium* [10].

In the genome of *P. rubens*, numerous genes encoding proteins with Zn(II)_2_Cys_6_ domains were identified [12]. However, to date, only two of these genes have been functionally characterized in this fungus. One of these genes, *sorR1*, is part of the sorbicillin BGC in *P. rubens*. Guzmán-Chávez et al. [13] demonstrated that *sorR1* positively regulates the expression of all the genes within this BGC. The second characterized gene is *nirA*, a well-known regulatory gene involved in nitrate assimilation in fungi [14]. Espeso et al. [15] examined a *P. rubens* strain with a natural mutation in *nirA*, resulting in a truncation of the NirA protein and an impaired nitrate assimilation. The subsequent complementation of this strain with a functional *nirA* gene led to an increase in the transcriptional levels of genes related to nitrate assimilation and improved growth of the fungus on nitrate-containing medium. To the best of our knowledge, no other gene encoding Zn(II)_2_Cys_6_ proteins has been functionally studied in *P. rubens*.

A few years ago, a gene named *pcz1* (for *Penicillium* C6 zinc domain protein 1), encoding a protein with a Zn(II)_2_Cys_6_ domain, was characterized in the cheese-ripening fungus *Penicillium roqueforti* [16]. In *P. roqueforti*, Pcz1 exerts significant effects on asexual development, including promotion of apical growth and conidiation, and repression of conidial germination [16,17]. Furthermore, *pcz1* exerts important effects on the production of the main specialized metabolites of *P. roqueforti* [17]. It is worth noting that orthologues of *pcz1* are widely distributed in fungi of the Ascomycota phylum, including *P. rubens* [16]. However, the functional role of this gene has only been experimentally investigated in *P. roqueforti* and remains unexplored in other fungi.

Recently, Pohl et al. [18] developed CRISPR/Cas9 tools for genetic modification in *P. rubens*, thus enabling the application of this technique in the fungus [19,20,21]. The authors established two CRISPR/Cas9 systems for *P. rubens*. One approach involved direct introduction of Cas9 protein and an in vitro synthesized sgRNA-ribonucleoprotein complex into fungal cells through transformation. The other approach involved transient transformation of the fungus with an AMA-based plasmid carrying cassettes for sgRNA and Cas9 expression within the fungal cells. The simplicity of the second approach makes it particularly attractive for gene disruption in the fungus. Consequently, in this study, we employed the second approach to disrupt the *pcz1* gene in *P. rubens* using CRISPR/Cas9. The functional characterization of the *pcz1*-disrupted mutants indicated that the gene acts as a positive regulator of penicillin BGC expression and penicillin production in *P. rubens*. Furthermore, *pcz1* exerts an important influence on the asexual development processes in the fungus, specifically controlling growth, conidiation, and germinal conidiation.

## 2. Materials and Methods

### 2.1. Fungal Strain and Culture Media

In this work, we used *P. rubens* Wis 54-1255, a strain widely used as a reference in molecular biology and OMICs studies [3]. The fungus was routinely kept on potato dextrose agar (PDA, Difco, Sparks, MD, USA) and grown at 28 °C. Various culture media were employed for phenotypic characterization, including Czapek, CYA, YES [22], Power [23], and CM [16]. The specific media utilized for penicillin production are described in the corresponding protocol (see below).

### 2.2. Selection of a Target Site for CRISPR/Cas9-Mediated Disruption of the pcz1 Gene

The sequence of the *pcz1* gene from *P. rubens* Wis 54-1255 (see Results) was used to select a target site using the CRISPR/Cas9 target prediction tool CCTop (https://cctop.cos.uni-heidelberg.de:8043, accessed on 18 August 2021) [24], which contains the genome sequence of *P. rubens* as a reference. CCTop was utilized with default parameters, except for mismatches considered during the prediction in the core sequence and the total number of mismatches, which were both set to 0. A predicted target sequence (5′ GGCCGGGCATGAGATCGACG 3′) with a high efficacy score (0.76) and located at the 5′ end of the coding sequence of the gene, was selected. To ensure specificity to the target sequence of interest and the absence of off-target effects, the selected sequence was aligned against the complete genome of *P. rubens* Wis 54-1255 using BLASTN.

### 2.3. Construction of Plasmid pFC333-Pcpcz1 for CRISPR/Cas9-Mediated Disruption of pcz1 in P. rubens Wis 54-1255

To achieve the disruption of *pcz1* in *P. rubens* Wis 54-1255 using CRISPR/Cas9, a plasmid named pFC333-Pcpcz1 was constructed. The plasmid construction involved the previous synthesis of an expression cassette by Integrated DNA Technologies (IDT, Coralville, IA, USA). The cassette was designed based on an expression cassette utilized in previous studies conducted on *Aspergillus* by Song et al. [25] and van Leewe et al. [26]. It comprised the promoter and gene sequence of the proline-tRNA (tRNA^Pro1^), the sgRNA (including the target sequence), and the terminator of tRNA^Pro1^. Additionally, a hammerhead ribozyme sequence was included between the tRNA^Pro1^ gene sequence and sgRNA, and *Pac*I restriction sites were added at the ends of the cassette.

The cassette synthesized was then digested with *Pac*I and cloned into plasmid pFC333 [27], previously digested with *Pac*I, thus giving rise to the final plasmid pFC333-Pcpcz1. Plasmid pFC333 contains the gene encoding Cas9 under the control of *tef1*-promoter from *A. nidulans*, the AMA region enabling autonomous replication of the plasmid, and the phleomycin resistance marker for selection of fungal transformants [27].

### 2.4. Transformation of P. rubens Wis 54-1255 and Obtainment of Transformants

The plasmid pFC333-Pcpcz1 was introduced into *P. rubens* Wis 54-1255 through protoplast transformation. Briefly, protoplasts were obtained using lytic enzymes (Sigma, St. Louis, MO, USA) and the transformation process followed the protocol established by Chávez et al. [28]. Transformants were selected on Czapek-sorbitol medium containing phleomycin, as described by Gil-Durán et al. [16]. Subsequently, conidia of the transformants underwent serial dilutions and were seeded onto phleomycin-containing Czapek medium to obtain homokaryotic strains. To induce the loss of phleomycin resistance, the homokaryotic strains were transferred four times to PDA medium without phleomycin (non-selective growth conditions). Confirmation of the loss of phleomycin resistance was achieved by observing the absence of growth of the transformants when plated again on selective media.

DNA was extracted following the method described by Gil-Durán et al. [16]. This DNA was used as a template for amplifying the target region using appropriate primers (Table 1). The resulting amplicons were then cloned in pGEM-T Easy and sent to Macrogen Inc. (Seoul, Republic of Korea) for sequencing.

### 2.5. Growth, Conidiation, and Conidial Germination Analyses

Apical extension rates were determined following the method described by Ivey et al. [29]. Briefly, 0.2 μL of a conidia suspension (10^6^ conidia/mL) was inoculated at the center of a Petri dish containing PDA, Czapek, CYA, YES, or Power medium. The plates were then incubated at 28 °C for 7 days. The diameters of the colonies were initially measured after 48 h and subsequently at 24 h intervals, with the outer white edge of the colonies serving as the reference point. To calculate the apical extension rate, a linear regression analysis was performed using the colony diameters plotted against time.

The production of conidia was measured following the methodology outlined by García-Rico et al. [30]. In brief, 100 μL of a conidial suspension (5 × 10^5^ conidia/mL) was spread onto Petri dishes containing either Czapek or Power media. The dishes were incubated at 28 °C for 3, 5, or 7 days, and the resulting conidia were collected by adding NT solution (0.9% NaCl, 0.05% Triton) and gently scraping the plate’s surface using an inverted Pasteur pipette. This procedure was repeated once, and the collected conidia were quantified using a Neubauer chamber. The conidia counts were expressed as conidia/mm^2^ of surface area.

Finally, conidial germination analysis was performed following the method described by Gil-Duran et al. [16]. Three replicate flasks containing CM or Czapek medium were inoculated with 2 × 10^5^ conidia/mL. The flasks were incubated at 28 °C for 12 h, and at regular intervals, 10 μL samples were collected from each flask. These samples were observed under a microscope, and the number of germinated and non-germinated conidia was counted in 10 randomly selected fields. This process was repeated twice for each flask to ensure technical replication. Conidia were classified as germinated when the length of their germ tubes equaled or exceeded the diameter of the conidia. The resulting data were plotted as the percentage of germination over time.

### 2.6. Production of Benzylpenicillin and HPLC Analyses

To produce benzylpenicillin, *P. rubens* was grown on Power medium agar [23] at 28 °C for 5 days. All the spores from a plate were collected and inoculated into 50 mL of *Penicillium* seed medium (MCIP) (sucrose 20 g/L, corn steep solids 20 g/L, yeast extract 10 g/L, CaCO_3_ 5 g/L, pH 5.7) [31]. The culture was incubated at 25 °C and 250 rpm for 24 h. Subsequently, 10 mL of this culture was inoculated into 100 mL of complex medium for fermentation of *Penicillium* (MCFP) (lactose 55 g/L, corn steep solids 35 g/L, CaCO_3_ 10 g/L, KH_2_PO_4_ 7 g/L, MgSO_4_·7H_2_O 3 g/L, potassium phenylacetate 4 g/L, pH 6.8) [31] and incubated under the same conditions for 48 and 72 h.

The extraction and quantification of benzylpenicillin from these broths were conducted as described by García-Estrada et al. [32]. In brief, 5 mL of the culture broth was centrifuged at 9000 rpm to separate the broth from the mycelium. The mycelium was stored for subsequent use (dry weight determination, see below), while the clarified broth (approximately 3 mL) was acidified to pH 2.0 using 0.1 N HCl. Benzylpenicillin was extracted from the broth three times by adding 2 mL of *n*-butyl acetate, and then the organic phase was re-extracted three times with 2 mL of 10 mM phosphate buffer at pH 7.5. The aqueous phase was lyophilized, resuspended in 1 mL of Milli-Q water, and analyzed using HPLC.

HPLC analysis was performed using a Waters system comprising a Waters 1525 Binary HPLC pump, a Waters 2996 Photodiode Array Detector, and an analytical 4.6 × 250 mm (5 μm) RPC18 SunFire^®^ 100 column (Waters, Wexford, Ireland). A flow rate of 1 mL/min and a detector wavelength of 214 nm were employed. Samples (50 μL) were injected and eluted using a mobile phase consisting of buffer A (30 mM ammonium formate pH 5.0 and 5% acetonitrile) and buffer B (buffer A plus acetonitrile 20:80, *v*/*v*) with an isocratic method (85% of A). In all analyses, pure commercial benzylpenicillin was used as standard.

Penicillin production was normalized based on the dry weight of the mycelium. For this purpose, the remaining mycelium was washed with 2 mL of a 1 M HCl solution, followed by centrifugation at 9000 g for 5 min. The supernatant was discarded, and the washed mycelium was then lyophilized, and its dry weight was determined.

### 2.7. qRT-PCR Analysis of the Expression of Penicillin Biosynthesis Genes

To isolate RNA, the mycelia were frozen in liquid nitrogen and ground in a mortar. Total RNA extraction was performed using the Plant/FungiTotal RNA Purification Kit (Norgen Biotek Corp., Thorold, ON, Canada) according to the manufacturer’s instructions. To eliminate any contaminating DNA, the extracted RNA was treated with the RNase-Free DNase I Kit (Norgen Biotek Corp, Thorold, ON, Canada). The concentration of RNA was determined using a MultiSkan GO quantification system with a μDrop plate (Thermo Fischer Scientific, Braunschweig, Germany). Next, 1 μg of total RNA was used to synthesize cDNA using All-In-One 5× RT MasterMix (Applied Biological Materials, Richmond, BC, Canada) according to the manufacturer’s instructions.

For gene expression analysis, qRT-PCR was conducted using the primers described in Table 1. Reactions were prepared in 20 μL volumes, containing 10 μL of KAPA SYBR Fast qRT-PCR Master Mix 2× (Kapa Biosystems, Wilmington, MA, USA), 0.4 μL of each primer (at a concentration of 10 μM each), 0.4 μL de 50× ROX High/Low, 6.8 μL of water, and 2 μL of cDNA. The quantification was performed on a StepOne Real-Time PCR System (Applied Biosystems, Waltham, MA, USA) with the following conditions: 30 s at 95 °C and 40 cycles of 3 s at 95 °C and 30 s at 60 °C. Negative controls were included in the experiments. Three replicates were performed for each gene expression analysis. Data were analyzed using the comparative Ct (2^−ΔΔCt^) method and were normalized to β-tubulin gene expression in each sample.

### 2.8. Phylogenetic Analysis of Pcz1

The Pcz1 protein sequence from *P. rubens* Wis 54-1255 was deduced from the corresponding gene sequence and subjected to BlastP analysis to identify sequences with high similarity. Multiple sequence alignment was performed using Clustal Omega, and a phylogenetic tree was constructed using MEGA version 7.0 [33]. The neighbor-joining method was employed to reconstruct the tree, and evolutionary distances were calculated using the Poisson correction model. To assess the robustness of the tree topology, bootstrap analyses with 1000 replications were conducted to estimate the support of internal nodes.

## 3. Results

### 3.1. Analysis of the pcz1 Gene and Deduced Protein from P. rubens Wis 54-1255

**Figure 1:**
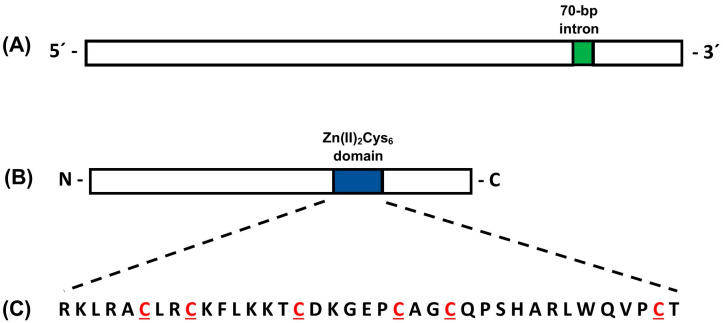
Schematic representations of the *pcz1* gene (**A**) and Pcz1 protein (**B**) from *P. rubens* Wis 54-1255. In panel (**A**), the single 70 bp intron is depicted in green. Panel (**B**) highlights the position of the Zn(II)_2_Cys_6_ domain in blue. Panel (**C**) shows the amino acid sequence of the Zn(II)_2_Cys_6_ domain, with the six conserved cysteines highlighted in red. The drawings are not to scale.

The *pcz1* gene from *P. roqueforti* [16] was utilized to scan the genome of *P. rubens* Wis 54-1255 using BLASTN and BLASTX. Both analyses yielded redundant results, confirming that the *pcz1* gene corresponds to the gene Pc22g12400 in the *P. rubens* Wis 54-1255 genome. The *pcz1* gene in *P. rubens* Wis 54-1255 spans 2446 bp and contains a single intron of 70 bp located at the 3′ end of the gene (Figure 1). This gene encodes a protein consisting of 791 amino acids. Analyses of conserved domains were performed using the HMMER tool at Interpro, and CDD at NCBI. Both analyses indicated the presence of a Zn(II)_2_Cys_6_ fungal-type domain spanning positions 394–431 of the Pcz1 protein, which contains the conserved six cysteines characteristic of such domains (Figure 1).

**Figure 2:**
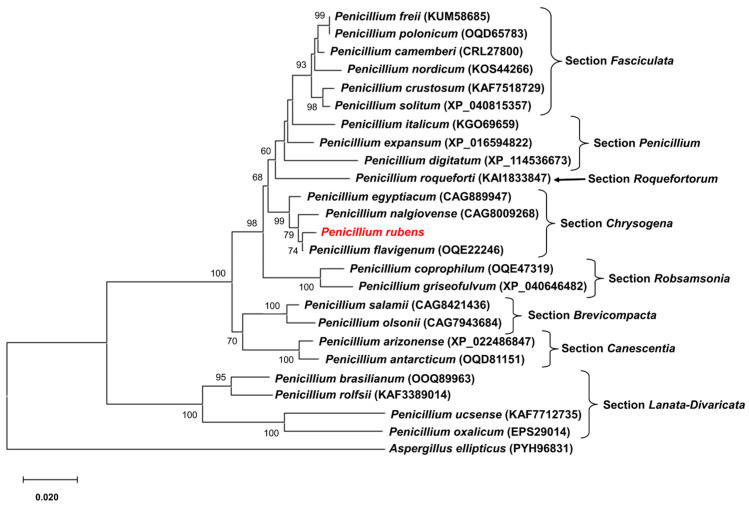
Phylogenetic tree depicting the relationships among Pcz1 proteins from *P. rubens* Wis 54-1255 and other fungal species. Pcz1 protein sequences from various species were obtained through BlastP analysis. The sequences included representatives from different sections within the *Penicillium* genus. The placement of the Pcz1 protein from *P. rubens* is highlighted in red. The GenBank accession number of each sequence is indicated in parentheses. The Pcz1 protein from *Aspergillus ellipticus* was used as an outgroup. Bootstrap values of 50% or higher are displayed at each node.

BlastP analysis of Pcz1 revealed the presence of closely related orthologues in various fungal species belonging to the phylum Ascomycota, particularly within the genus Penicillium. The evolutionary relationships between Pcz1 from P. rubens Wis 54-1255 and its closest orthologous proteins are depicted in Figure 2. This analysis showed that Pcz1 clustered with orthologues within section Chrysogena in Penicillium, in agreement with the established species phylogeny [1].

### 3.2. Generation of pcz1-Disrupted Strains of P. rubens Wis 54-1255 by CRISPR-Cas9

**Figure 3:**
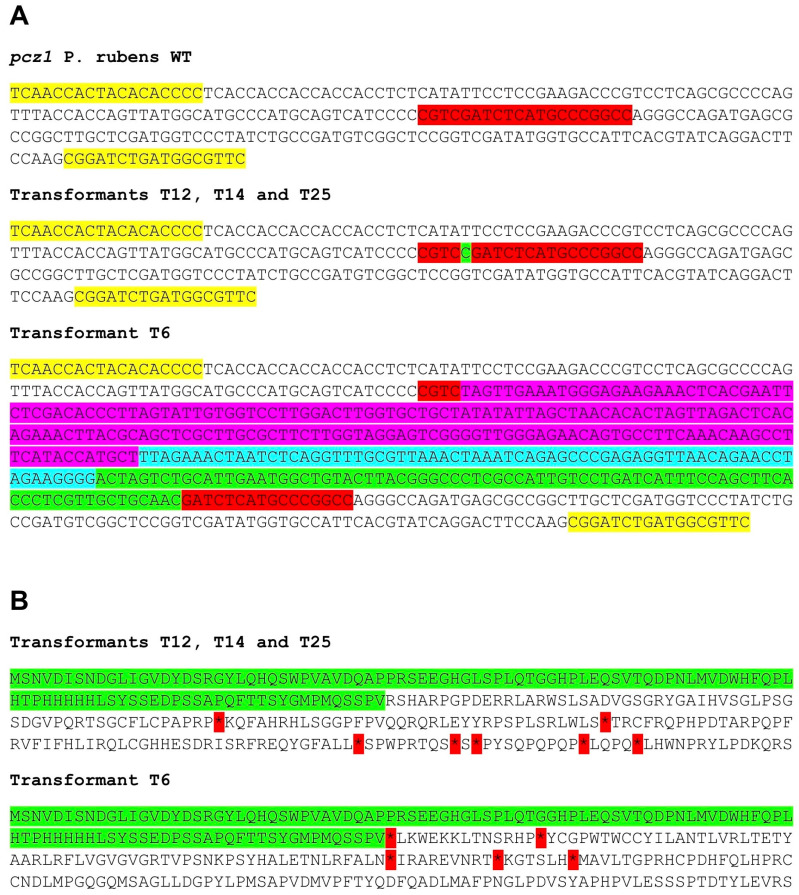
(**A**) Sequences of the target region in the *pcz1* gene from *P. rubens* wild type (WT) and transformants T6, T12, T14, and T25. The target sequence is highlighted in red, while the yellow region represents the primers used for amplification (see Table 1). Transformants T12, T14, and T25 exhibit the same one-nucleotide insertion (highlighted in green), while T6 displays different inserted segments from plasmid pFC333 (highlighted in fuchsia, turquoise, and green). (**B**) Deduced protein sequence of the target region from transformants T6, T12, T14, and T25. The unaltered protein sequence is highlighted in green. After this region, frameshifts occur in transformants, resulting in changes to the protein sequence and the generation of multiple stop codons (asterisks highlighted in red).

After the transformation of *P. rubens* Wis 54-1255 with plasmid pFC333-Pcpcz1, thirty-five transformants were obtained. These transformants underwent passages in Czapek media containing phleomycin, followed by passages in phleomycin-free Czapek media. Ten transformants lost phleomycin resistance, and their target region was amplified and sequenced. Notably, four of these transformants exhibited insertions in the *pcz1* gene, resulting in gene disruption due to the generation of multiple stop codons (Figure 3). Specifically, three transformants (T12, T14, and T25) shared the same 1 bp insertion, while transformant T6 displayed a large insertion of 336 bp, encompassing various fragments from the AMA region of plasmid pFC333 (Figure 3). Based on these findings, transformants T6 and T14, along with the wild-type strain, were selected for subsequent experiments.

### 3.3. Morphological Features of the pcz1-Disrupted Strains of P. rubens Wis 54-1255

**Figure 4:**
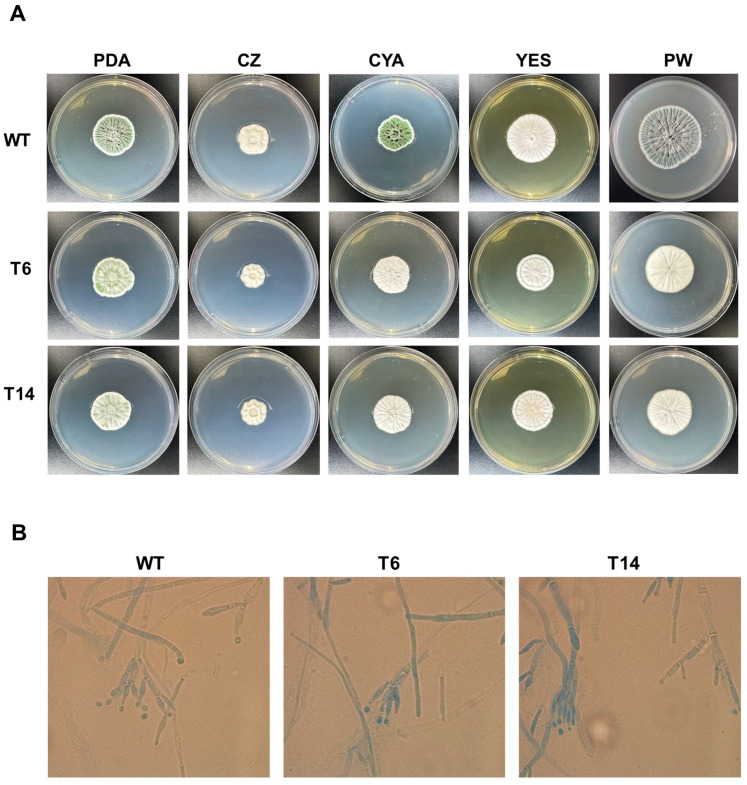
Phenotypic characteristics of *P. rubens* strains. (**A**) Colony morphology of *P. rubens* Wis 54-1255 (WT) and transformants T6 and T14 after 7 days of growth at 28 °C on PDA, Czapek (CZ), CYA, YES and Power (PW) media. (**B**) Microscopic observation of *P. rubens* Wis 54-1255 (WT) and transformants T6 and T14. Fungal samples were obtained from colonies grown on PDA agar for 7 days at 28 °C. Samples were stained with lactophenol cotton blue and observed under bright-field microscopy at 100× magnification.

As an initial approach to assess the phenotypic characterization of the transformants T6 and T14, they were subjected to examination in five different media and compared to *P. rubens* Wis 54-1255 grown under the same conditions (Figure 4A). In general, two discernible morphological changes were noted. Firstly, the characteristic green pigmentation associated with normal sporulation in *P. rubens* Wis 54-1255 was attenuated in transformants T6 and T14 in certain media, resulting in a visibly paler or even white aspect. This phenomenon was particularly pronounced in PDA, CYA, and Power media (Figure 4A). Additionally, the transformants displayed subtle differences in growth compared to the wild-type strain (Figure 4A), suggesting a slight reduction in their growth rate of the transformants. Both conidiation and growth rates will be examined in detail in subsequent experiments.

Furthermore, a microscopic examination of the strains was conducted (Figure 4B). All strains exhibited similar morphological characteristics under microscopic analysis, with no evidence of major morphological alterations. Thereby, hyphae displayed their typical elongated, branch-like structures characteristic of hyphal growth, while conidiophores exhibited the distinctive brush-like appearance typical of *Penicillium*.

### 3.4. The Disruption of pcz1 Reduces Growth and Conidiation, but Promotes Conidial Germination in P. rubens Wis 54-1255

**Figure 5:**
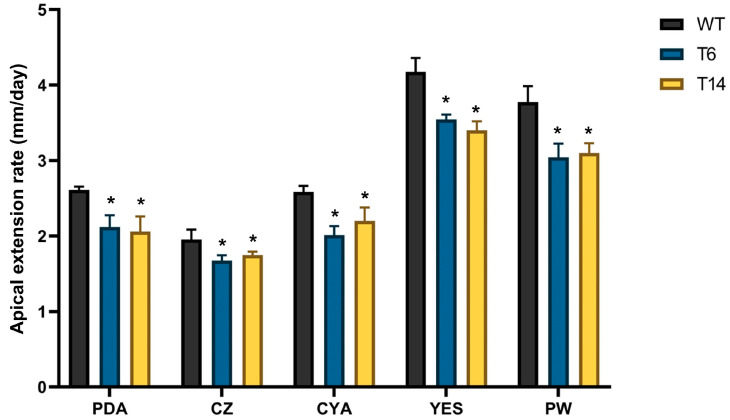
Apical extension rates of *P. rubens* Wis 54-1255 (WT) and transformants T6 and T14 on PDA, Czapek (CZ), CYA, YES, and Power (PW) media. Strains were incubated at 28 °C for 7 days. Error bars represent the standard deviation of three replicates in three different experiments. The symbol * indicates statistically significant differences (*p* < 0.05 using Student’s *t*-test) in apical extension rates compared to the wild-type strain in the respective medium.

The apical growth of fungal strains was evaluated in five different media. In all cases, the *pcz1*-disrupted strains T6 and T14 exhibited a slight delay in apical growth compared to the wild-type strain of *P. rubens* (Figure 5). Depending on the specific media used, transformants displayed a growth rate ranging between 77.6% and 89.7% of the wild-type fungus, indicating that *pcz1* acts as a positive regulator of growth in *P. rubens* Wis 54-1255.

**Figure 6:**
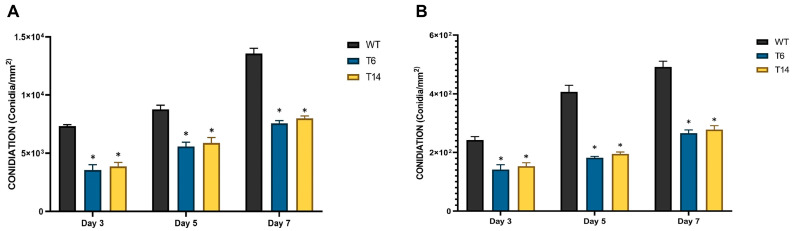
Conidia production of *P. rubens* Wis 54-1255 (WT) and transformants T6 and T14 in Power (**A**) and Czapek (**B**) media grown at 28 °C for 3, 5, and 7 days. Error bars represent the standard deviation of three replicates in three independent experiments. The asterisk (*) indicates statistically significant differences (*p* < 0.05 using Student’s *t*-test) in conidia production between the transformants and the wild-type strain.

Regarding conidiation, it was assessed in two different media: the minimal medium Czapek, and the nutrient-rich medium Power, specifically designed to enhance conidiation [23]. In both media, the *pcz1*-disrupted strains T6 and T14 displayed a significant decrease in conidia production compared to the wild-type strain (Figure 6). Depending on the specific media used and day of measurement, the transformants produced between 44.8% and 63.2% of the conidia produced by the wild-type fungus. These findings support the role of *pcz1* as a positive regulator of conidiation in *P. rubens* Wis 54-1255.

**Figure 7:**
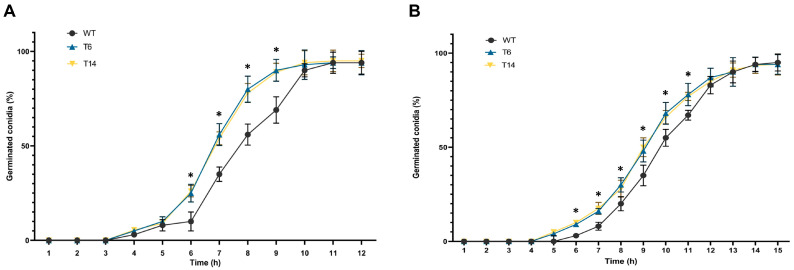
Germination rates of *P. rubens* Wis 54-1255 (WT) and transformants T6 and T14 represented as the percentage of germinated conidia versus hours of incubation in CM-rich medium (**A**) and Czapek minimal medium (**B**). Error bars represent the standard deviation of three replicates from three independent experiments. The asterisks (*) indicate points on the curves where statistically significant differences (*p* < 0.05 using Student’s *t*-test) were observed between the transformants and the wild-type strain.

Finally, conidial germination was assessed. As shown in Figure 7, the *pcz1*-disrupted strains T6 and T14 exhibited earlier conidial germination compared to the wild-type strain. For instance, after 7, 8, and 9 h in CM medium, the transformants displayed approximately 56%, 80%, and 90% of conidia germinated, respectively. In contrast, the wild-type *P. rubens* Wis 54-1255 exhibited significantly lower values of 35%, 56%, and 69% of conidia germinated at the same time points and in the same medium. A similar trend was observed in the minimal medium Czapek, although the differences were less pronounced. These findings indicate that *pcz1* functions as a negative regulator of conidial germination in *P. rubens* Wis 54-1255.

### 3.5. The Inactivation of pcz1 Reduces the Production of Penicillin and the Expression of the Penicillin Gene Cluster in P. rubens Wis 54-1255

**Figure 8:**
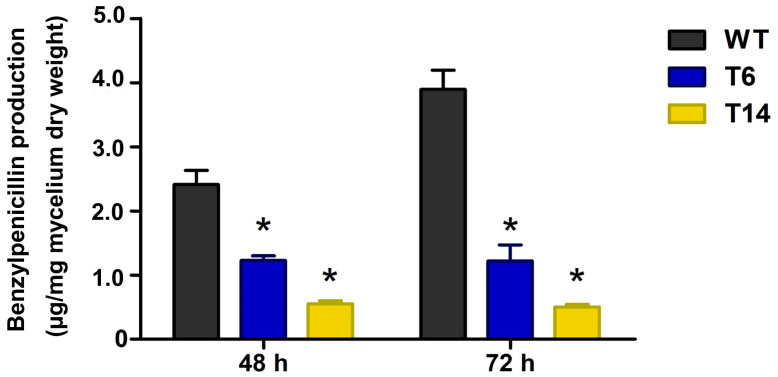
Production of penicillin by *P. rubens* Wis 54-1255 (WT) and transformants T6 and T14 in MCFP medium at 48 and 72 h. Data are average of three replicas from three different experiments. Error bars indicate the standard deviation of the mean value. The symbol * indicates statistically significant differences of transformants (*p* < 0.05 using Student’s *t*-test) as compared to the wild-type strain at the same times.

Penicillin is the most important specialized metabolite produced by *P. rubens*; therefore, we studied its production in the transformants. As depicted in Figure 8, the production of benzylpenicillin was significantly reduced in the *pcz1*-disrupted strains. While the wild-type strain of *P. rubens* Wis 54-1255 produced an average of 2.4 and 3.9 μg/mg of penicillin at 48 and 72 h, respectively, transformant T6 produced 1.2 μg/mg of penicillin, and transformant T14 produced 0.6 and 0.5 μg/mg of penicillin at the same time points (Figure 8). These results indicate that the inactivation of *pcz1* decreases the production of benzylpenicillin, suggesting that this gene exerts a positive regulation on the production of this important specialized metabolite in *P. rubens* Wis 54-1255.

**Figure 9:**
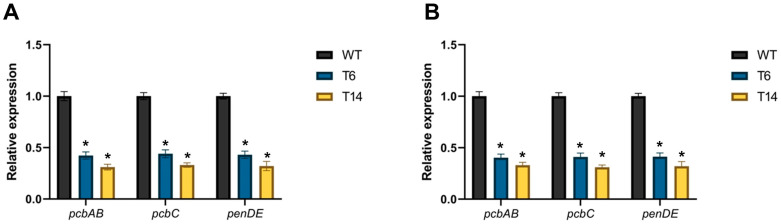
Relative expression of *pcbAB*, *pcbC*, and *penDE* in *pcz1*-disrupted transformants at 48 h (**A**) and 72 h (**B**) compared to the control *P. rubens* wild-type strain (WT). The data represent the mean values from three separate experiments, each consisting of three replicates. The error bars indicate the standard deviation of the mean. The symbol * indicates statistically significant differences between transformants and the wild-type strain (*p* < 0.05, assessed using Student’s *t*-test).

Finally, the impact of *pcz1* disruption on the expression of the genes in the penicillin BGC was investigated. For this purpose, the relative expression of *pcbAB*, *pcbC*, and *penDE* genes was measured. As shown in Figure 9, the relative expression of these three genes in the *pcz1*-disrupted strains was significantly lower compared to the wild-type strain. Transformants T6 and T14 exhibited an important reduction in *pcbAB* transcripts, with a 2.4- and 3.2-fold decrease at 48 h, and a 2.5- and 3.0-fold decrease at 72 h, respectively, compared to *P. rubens* Wis 54-1255. Similarly, *pcbC* transcripts showed a 2.3- and 3.0-fold decrease at 48 h, and a 2.4- and 3.2-fold decrease at 72 h, respectively, in the disrupted strains. And in the case of *penDE*, transcripts displayed a 2.3- and 3.1-fold decrease at 48 h, and a 2.4- and 3.1-fold decrease at 72 h, respectively. These findings confirm that Pcz1 positively regulates the expression of penicillin biosynthetic genes.

## 4. Discussion

To date, the role of the *pcz1* gene has only been investigated in the cheese-ripening fungus *P. roqueforti*. In this organism, it has been observed that *pcz1* acts as a positive regulator of growth and conidiation, while exerting a negative regulation on conidial germination [16]. The results obtained in our study indicate that the role of *pcz1* in the regulation of these processes is conserved in *P. rubens*. Taken together, these findings suggest an emerging role of *pcz1* in asexual development across *Penicillium* species, possibly through conserved mechanisms, and underscore the importance of further exploring the regulatory role of *pcz1* in fungi beyond the *Penicillium* genus.

In addition to its impact on asexual development, the inactivation of *pcz1* in *P. rubens* Wis 54-1255 has an important effect on penicillin production. Specifically, the *pcz1*-disrupted strains T6 and T14 exhibited a significant reduction in benzylpenicillin production compared to the wild-type strain. The decrease in penicillin production ranged from approximately 50% to 87% in comparison to the wild-type strain at 48 and 72 h, respectively (Figure 8). These results provide clear evidence that the inactivation of *pcz1* diminishes the production of penicillin, indicating a positive regulatory role of *pcz1* in the biosynthesis of this important specialized metabolite in *P. rubens* Wis 54-1255. To the best of our knowledge, this is the first study implicating a gene encoding a Zn(II)_2_Cys_6_ protein in the regulation of penicillin biosynthesis in *P. rubens*.

The positive regulatory role of *pcz1* in penicillin production suggests that the overexpression of this regulator could potentially serve to increase penicillin production in *P. rubens*. This hypothesis finds support in recent research demonstrating that overexpression of *pcz1* in *P. roqueforti* increased the production of mycophenolic acid, an important immunosuppressive compound [17]. In *P. rubens*, various efforts have been made to increase penicillin production through the overexpression of global regulators, yielding varied results. For instance, the overexpression of regulators like PcLaeA, PcRFX1, PcYap1, PcRsmA, and PcFKH1 led to an increased production of penicillin, although in some cases (for example, PcFKH1) the increase was modest [34,35,36,37]. Conversely, the overexpression of the positive regulators of penicillin production StuA and MAT1-1-1 had no impact on penicillin titers in *P. rubens* [38,39]. Thus, the effectiveness of employing the overexpression of global regulators to enhance penicillin production in *P. rubens* varies on a case-by-case basis. In the future, it will be interesting to investigate whether the overexpression of *pcz1* effectively increases penicillin production in *P. rubens*.

In *P. roqueforti*, Rojas-Aedo et al. [17] employed RNAi-mediated gene silencing to downregulate the expression of *pcz1* and measured the effect of this genetic manipulation on the production of three specialized metabolites: roquefortine C, andrastin A, and mycophenolic acid. They observed reduced production of the three specialized metabolites in the RNAi-downregulated strains, which correlated with decreased transcription of genes within the biosynthetic gene clusters responsible for their biosynthesis [17]. Collectively, our results and these previous findings in *P. roqueforti* suggest a potential general role for *pcz1* in regulating specialized metabolite production in fungi.

The precise molecular mechanism by which Pcz1 exerts its regulatory role remains unknown. As mentioned in the Introduction, Pcz1 is a protein containing a Zn(II)_2_Cys_6_ domain, a domain unique to fungi that is commonly found in “zinc finger” proteins [10,40]. Notable examples of fungal proteins containing this kind of domain include Gal4, a transcription factor involved in galactose metabolism in yeast [41], and AflR, a regulator of aflatoxin biosynthesis in *Aspergillus* [42]. These proteins typically bind specific DNA promoter sequences of their target genes [41,42]. Therefore, it is plausible to hypothesize that Pcz1 exerts its regulatory function by directly or indirectly interacting with promoter sequences of one or more target genes. Further investigations, such as chromatin immunoprecipitation coupled with DNA sequencing, are necessary to elucidate the putative binding sequences of Pcz1 in promoter regions. These experimental approaches are clearly beyond the scope of the present study.

A recent review proposed a regulatory model of *pcz1* based on findings in *P. roqueforti* [43]. According to this model, the expression of *pcz1* is negatively influenced by *pga1*, a gene that encodes a heterotrimeric G protein alpha subunit [43]. The effects of Pga1 on asexual development in *P. rubens* have been previously investigated [30], and these findings match well with the proposed model for *P. roqueforti* and the results obtained in our study. However, regarding penicillin production, the regulatory model of Pga1 on Pcz1 proposed for *P. roqueforti* does not agree with the experimental evidence in *P. rubens*. According to the model, a negative regulatory role of Pga1 on Pcz1 in *P. rubens* would imply a negative regulation of penicillin production by Pga1, contradicting previous experimental data that indicate a positive effect of Pga1 on penicillin production in *P. rubens* [44]. Thus, the relationship between Pga1 and Pcz1 appears to be more complex than the model proposed for *P. roqueforti*, and it is possible that the relationship between Pga1 and Pcz1 differs between *P. rubens* and *P. roqueforti*. This highlights the importance of conducting investigations to understand the regulation of fungal specialized metabolism on a case-by-case basis, as the functional relationships observed in one fungal species may not necessarily apply to others.

In conclusion, our study provides evidence for the significant role of Pcz1, a protein containing a Zn(II)_2_Cys_6_ domain, in governing asexual development and functioning as a regulator of penicillin production in *P. rubens*. It is important to note that penicillin production in this fungus is controlled by a complex network of regulatory circuits that are not yet fully understood [3]. Pcz1 emerges as a novel component within this regulatory network, and the future study of its mechanisms will contribute to improving our understanding of the regulation of penicillin production.

## Figures and Tables

**Table 1:** Primers used in this work.

Name of the Primer	Sequence (5′---3′)	Used for:
Conf-Pcz1-CRISPR-FW Conf-Pcz1-CRISPR-RV	TCAACCACTACACACCCC GAACGCCATCAGATCCG	Amplification of the target sequence of the *pcz1* gene
qPCR-pcbAB-fw qPCR-pcbAB-rv	ACGACAACTTCTTCCGCCTA AGATGCTGACCGAGAGTCGT	*pcbAB* gene expression analysis by qRT-PCR
qPCR-pcbC-fw qPCR-pcbC-rv	GACGTGTCGCTCATTACCGT AATTGACCAGGTAGGCGTTG	*pcbC* gene expression analysis by qRT-PCR
qPCR-penDE-fw qPCR-penDE-rv	CATCCTCTGTCAAGGCACTCC CCATCTTTCCTCGATCACGC	*penDE* gene expression analysis by qRT-PCR
qRT-btub-fw qRT-btub-rv	TCCAAGGTTTCCAGATCACC GAACTCCTCACGGATCTTGG	β-tubulin gene expression analysis by qRT-PCR

## Data Availability

Not applicable.
